# Predictive Factors for Long-Term Outcomes of Cataract Surgery in Patients Receiving Active Treatment for Neovascular Age-Related Macular Degeneration

**DOI:** 10.3390/jcm10143124

**Published:** 2021-07-15

**Authors:** Eun Young Choi, Tae Young Kim, Christopher Seungkyu Lee

**Affiliations:** 1 Department of Ophthalmology, Severance Eye Hospital, Yonsei University College of Medicine, 50-1, Yonseiro, Seodaemun-gu, Seoul 03722, Korea; eunyoung.choi86@gmail.com; 2 Department of Ophthalmology, Gangnam Severance Hospital, Yonsei University College of Medicine, 211, Eonjuro, Gangnam-gu, Seoul 06273, Korea; ANDREWTY2@yuhs.ac

**Keywords:** neovascular age-related macular degeneration, cataract surgery, anti-vascular endothelial growth factor therapy, visual acuity, predictive factors

## Abstract

Background: the safety and efficacy of cataract surgery in eyes with exudative neovascular age-related macular degeneration (nAMD), receiving active treatment, remain unclear. We evaluated the long-term outcomes and associated predictive factors of cataract surgery in eyes with exudative nAMD. Methods: this retrospective cohort study included 65 eyes (61 patients) treated with anti-vascular endothelial growth factor (VEGF) injections within six months preoperatively. Changes in best-corrected visual acuity (BCVA) and anti-VEGF treatment patterns from before to up to four years after surgery were assessed. Predictive factors were identified in association with one-year surgical outcomes. Results: the BCVA improved at six months (*p* < 0.001) and was maintained for three years postoperatively. The interval between anti-VEGF injections increased 3.4 times postoperatively (*p* = 0.001). Risk factors for poor BCVA were low preoperative BCVA (*p* < 0.001) and prolonged nAMD duration (*p* = 0.003). Prolonged nAMD duration and short exudation-free period were associated with more frequent postoperative anti-VEGF treatments (*p* = 0.028 and *p* = 0.003, respectively). AMD subtypes were not associated with both vision and injection pattern outcomes. Conclusions: patients with cataracts receiving nAMD treatment can safely undergo surgery with favorable long-term visual benefits. The preoperative BCVA, nAMD duration, and exudation-free period are potential predictors of surgery outcomes.

## 1. Introduction

Cataracts and age-related macular degeneration (AMD) are the leading causes of vision impairment, especially in older patients [1,2]. The prevalence of these conditions will increase proportionally in an aging global population. Advancements in cataract surgery have facilitated the cost-effective and successful treatment of cataracts [3], and the advent of anti-vascular endothelial growth factor (VEGF) agents has dramatically improved the outcome of neovascular AMD (nAMD) treatment [4,5].

The effects of cataract surgery on non-exudative AMD have been investigated extensively in previous cross-sectional studies [6], randomized clinical studies [7,8,9,10], and meta-analyses [11,12]. Most of these studies reported no significant association between cataract surgery and the progression of non-exudative AMD. The safety and efficacy of cataract surgery in patients with exudative nAMD have also been investigated, although in a limited number of non-comparative [13,14,15,16,17,18] and comparative studies [19,20], conducted in patients with nAMD who had and had not undergone cataract surgery. Most studies have shown that cataract surgery is beneficial for vision improvement without exacerbating the progression of AMD. However, some of these studies had notable limitations. Two non-comparative studies only included approximately 24–28% of the exudative status of patients with AMD at the time of surgery [15,18]. In other studies, the participants were not administered anti-VEGF (stable neovascular complex) agents in the six months prior to surgery, or their preoperative exudation-free periods were not described in detail [13,15,17]. Moreover, the follow-up durations of these studies were limited to three months to one year postoperatively [16,18,19].

A recent longitudinal study that analyzed data from a health insurance database showed that cataract surgery is associated with an increased risk of nAMD [21]. The long-term outcomes after cataract surgery, especially in the eyes of patients on active treatment for nAMD, are still largely unknown.

The overall short-term safety of cataract surgery in patients with nAMD has been confirmed; however, for patients receiving active treatment for nAMD, specific preoperative factors that predict poor visual prognosis and recurring or worsened exudation remain unclear. One study found that a short AMD duration and a long exudation-free period were associated with reduced exudation after cataract surgery [17]. However, the predictors for recently treated nAMD eyes are still unknown since this study only included exudation-free eyes with nAMD.

The safety and efficacy of cataract surgery in eyes with nAMD receiving active treatment remain unclear. Knowledge of the long-term outcomes and factors predicting them would be of immense value for clinicians treating patients with visually significant cataracts and exudative nAMD. Therefore, we conducted this study to determine the three-year outcomes of cataract surgery and to identify the prognostic factors related to the progression of exudative nAMD in eyes receiving active anti-VEGF therapy.

## 2. Materials and Methods

### 2.1. Study Design

This retrospective, observational cohort study used data extracted from the medical records of the Severance Hospital of Yonsei University College of Medicine (a tertiary referral center) at Sinchon and Gangnam, Seoul, Korea. Ethical approval was obtained from the Institutional Review Board of Gangnam Severance Hospital (approval number, 3-2019-0378). The requirement for patient consent was waived because patient data were anonymized. This study was conducted in adherence to the tenets of the Declaration of Helsinki.

### 2.2. Study Population

Patients who underwent uneventful cataract surgery between 1 July 2006 and 31 August 2019 while receiving anti-VEGF therapy for nAMD were considered for inclusion in this study. The eyes of patients who were followed up for at least 12 months after cataract surgery were included in the analysis. Only the eyes that received the last anti-VEGF injection within six months before cataract surgery were selected.

We identified 277 eyes suitable for inclusion from 304 patients with AMD who underwent cataract surgery; 82 eyes with no confirmed macular neovascularization before surgery were excluded. Furthermore, 72 eyes were excluded due to other ophthalmic co-morbidities (e.g., vitreous and submacular hemorrhage, retinal detachment, or retinoschisis) or with a history of any intraocular surgery (e.g., anterior vitrectomy, pars plana vitrectomy, or glaucoma surgery) that could affect vision. We also excluded 14 eyes that were followed up for less than one year after surgery, while we had no limit for the preoperative follow-up time. Moreover, we excluded 31 eyes that had received the last anti-VEGF injection more than six months before surgery and 13 eyes that had received anti-VEGF injections within an average interval of 6 months.

The primary outcome was analyzed in 65 eyes of 61 patients. Four patients had bilateral AMD, and both eyes were included in the analyses. Among the included eyes, 43 had been followed up for at least one year pre- and postoperatively, as required for comparing the frequency of anti-VEGF injections.

### 2.3. Measurements and Outcomes

In addition to demographic data, we recorded the dates of nAMD diagnosis, cataract surgery, and all anti-VEGF injections before and after surgery. We also investigated the detailed history of anti-VEGF therapy (duration, type of drug used, injection regimen, number of injections, and interval between injections) and cataract surgery (date, surgical method, and combined procedures, such as anti-VEGF injection and anterior vitrectomy). Injection regimens were divided into two: pro re nata (PRN) and treat-and-extend (TAE). The baseline was defined as the time when the first anti-VEGF injection for nAMD treatment was administered. The period before cataract surgery was referred to as “preoperative.” The injections combined with cataract surgery were considered as “postoperative” treatment.

We evaluated the presence of exudation indicators (collection of fluid or hemorrhage in the intraretinal or subretinal spaces) depending on the findings of optical computed tomography (OCT). “Exudation-free” was defined as completely dry, with no exudation indicators. The exudation-free period before cataract surgery was measured in days. All eyes treated with the PRN regimen were included to evaluate the interval to the subsequent recurrence; in the TAE regimen, we considered only those with exudation confirmed on OCT at the first postoperative injection.

The primary outcome was the change in visual acuity (VA) at six months, one year, and three years after cataract surgery. The secondary outcome was the identification of postoperative factors that could predict the visual outcome. Variables considered in the analyses of predictive factors included patient age, type of AMD lesion, the interval between first and last intravitreal anti-VEGF injection and cataract surgery, preoperative exudation-free period, and the presence of exudation during surgery.

### 2.4. Statistical Analysis

Descriptive data are presented as mean ± standard deviation (SD) or frequencies (with percentages), as appropriate. A paired *t*-test was used to analyze differences before and after cataract surgery. We used simple regression analysis with a linear mixed-effect model to evaluate the relationship between each prognostic factor and the visual outcome and frequency of anti-VEGF injections. Subsequently, we included the predictive prognostic factors with the lowest *p*-values in a multiple regression model, created using a stepwise backward selection procedure. All statistical analyses were conducted using SPSS software version 21.0 (IBM, Armonk, NY, USA). A *p*-value < 0.05 was considered to indicate statistical significance.

## 3. Results

### 3.1. Demographic and Clinical Characteristics

The patients’ demographic characteristics are summarized in Table 1. The initial mean ± SD best-corrected visual acuity (BCVA) was 0.71 ± 0.45 logarithm of the minimum angle of resolution (logMAR), and the mean BCVA before cataract surgery was 0.91 ± 0.45 logMAR. The average time between the diagnosis of nAMD and cataract surgery was 32.7 ± 32.9 months (range: 4–132). PRN injections were administered in 43 eyes (66.2%), while 22 eyes (33.8%) were treated under the TAE regimen. Three cases treated with the PRN regimen preoperatively switched to the TAE regimen after surgery, and no case changed from TAE to PRN. The mean number of anti-VEGF injections administered during the preoperative period was 14.2 ± 13.6.

Before cataract surgery, 20 eyes (30.8%) were treated with bevacizumab, 29 (44.6%) with ranibizumab, and 16 (24.6%) with aflibercept. The average interval between the last anti-VEGF injection and cataract surgery was 70.8 ± 55.8 days. The presence of exudative fluid was confirmed using preoperative OCT examinations in 46 of 65 eyes (70.8%). A total of 33 of 65 eyes (50.8%) received anti-VEGF injection combined with cataract surgery. Among these eyes, 30 (90.9%) were originally scheduled for injection at approximately the time of cataract surgery, and three (9.1%) received an additional injection to protect against cataract surgery.

### 3.2. Long-Term Changes in VA after Cataract Surgery

**Figure 1:**
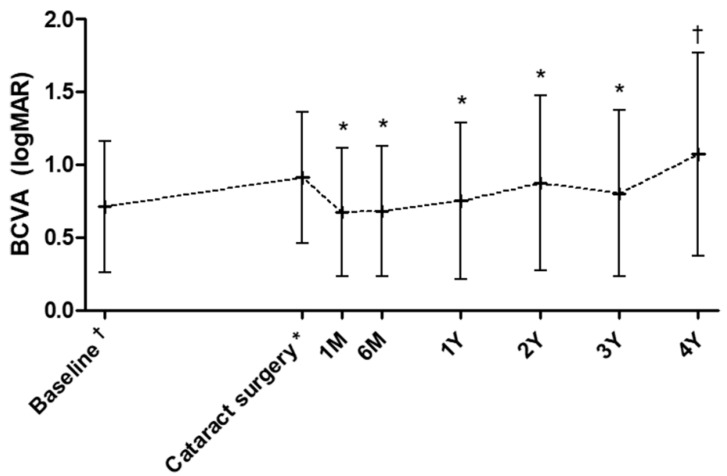
Changes in the mean best-corrected visual acuity from an average of 32.7 months before surgery (baseline), at the time of cataract surgery, and follow-ups. Number of patients at each time point: 65 (6M), 65 (1Y), 61 (2Y), 51 (3Y), and 39 (4Y). Values are expressed as mean ± standard deviation (error bars). Statistical significance on a paired t-test (*p* < 0.05) when compared with the preoperative point is indicated by the asterisks (*) over the bars and when compared with baseline by the cross (†). M: months, Y: year(s), BCVA: best-corrected visual acuity, logMAR: logarithm of the minimum angle of resolution.

The change in BCVA after cataract surgery is shown in Figure 1. The BCVA significantly improved six months (*n* = 65; 0.68 logMAR; *p* < 0.001) and one year (*n* = 65; 0.75 logMAR; *p* = 0.001) postoperatively compared to the preoperative BCVA (0.91 logMAR). This improvement in vision was maintained for up to three years postoperatively (*n* = 51; 0.87 logMAR; *p* = 0.017).

The difference between the preoperative and postoperative BCVA was not significant four years after cataract surgery (*n* = 39; 1.07 logMAR; *p* = 0.34). The postoperative BCVA did not show any significant difference from the baseline BCVA at the time of nAMD diagnosis up to three years after cataract surgery (*p* = 0.81 for six months, *p* = 0.94 for one year, and *p* = 0.46 for three years postoperatively). However, the BCVA significantly decreased four years postoperatively, compared to the baseline BCVA (*p* = 0.044).

### 3.3. Changes in Treatment Patterns after Cataract Surgery

The mean interval to the subsequent postoperative injection was 88.1 days, and there was no significant difference when comparing the mean interval from the previous last injection to the time of surgery (70.8 days, *p* = 0.21; Table 1). The average exudation-free period increased significantly after surgery in the PRN regimen group (preoperative: 21.3 ± 10.4 days; postoperative: 112.9 ± 18.5; *p* = 0.004) and in the TAE regimen group (preoperative: 26.3 ± 12.5 days; postoperative: 65.9 ± 16.4; *p* = 0.033). The average interval between injections increased significantly after surgery (preoperative: 66.5 days; postoperative: 228.0 days; *p* = 0.001, Table 1). The average number of injections per year decreased significantly from 5.2 before surgery to 1.6 after surgery (*p* = 0.001). The mean injection interval significantly increased in the PRN regimen group (preoperative: 66.6 ± 34.3 days between injections; postoperative: 171.0 ± 15.7 days; *p* = 0.001), and approached significance in the TAE regimen group, with approximately half of the subjects (preoperative: 66.8 ± 44.7 days; postoperative: 234.3 ± 34.0 days; *p* = 0.064).

### 3.4. Predictive Factors for VA after Cataract Surgery

The results of simple and multiple linear regression analyses of prognostic factors affecting BCVA one year after surgery are presented in Table 2. Higher preoperative BCVAs were significantly associated with favorable VA outcomes (coefficient [CE]: 0.50; *p* < 0.001). A shorter AMD duration was also associated with a more favorable postoperative VA (CE: 0.004; *p* = 0.003).

Other factors had no significant predictive effects, including age (*p* = 0.76), AMD lesion subtype (*p* = 0.48), preoperative exudation-free period (*p* = 0.61), presence of exudation at surgery (*p* = 0.22), injection interval before surgery (*p* = 0.39), combined injection during cataract surgery (*p* = 0.11), and subsequent recurrence of exudation postoperatively (*p* = 0.22).

### 3.5. Predictive Factors for Anti-VEGF Injection Frequency after Cataract Surgery

Table 3 summarizes the results of simple and multiple linear regression analyses of prognostic factors affecting the interval between anti-VEGF treatments after cataract surgery. A longer AMD duration before cataract surgery was associated with an increased frequency of injections afterward (CE: −3.38; *p* = 0.028). A prolonged exudation-free period was associated with less frequent injections postoperatively (CE: 2.77; *p* = 0.003).

Other factors had no discernible predictive effects, including age (*p* = 0.49), AMD lesion subtype (*p* = 0.14), the presence of exudation at surgery (*p* = 0.18), frequency of injections before surgery (*p* = 0.17), combined injection during cataract surgery (*p* = 0.67), and subsequent recurrence of exudation after surgery (*p* = 0.71).

## 4. Discussion

We analyzed the outcomes of cataract surgery in eyes receiving active anti-VEGF treatment for exudative nAMD. The postoperative VA recorded six months after cataract surgery improved by 0.23 logMAR (0.12 Snellen equivalent) compared to the preoperative VA; this improvement was maintained until the three-year follow-up. The mean interval between anti-VEGF injections increased approximately 3.4 times postoperatively, and the mean exudation-free period almost quadrupled. These postoperative changes were more pronounced in the PRN regimen group compared to the TAE regimen group. The change in injection frequency may coincide with the long-term natural course of nAMD after treatment [22]. A low preoperative VA and long AMD duration were significant risk factors for a poor visual outcome. The preoperative AMD duration and the exudation-free period showed a significant association with the increased frequency of postoperative anti-VEGF injections.

The eyes included in this study were being actively treated with anti-VEGF injections at an interval of approximately two months before cataract surgery; this interval is relatively shorter than that reported by previous studies [13,15,16,17,19,20]. The presence of exudation was confirmed with OCT in 46 (70.8%) eyes included in this study, representing a higher proportion of the total sample than previously reported [15,18]. In these cases, the exudation-free period was considered zero, and the average exudation-free period (21.3 days) was much shorter than the average interval between injections (66.5 days). This difference appears because about half of the subjects (45.9%) have an average interval between injections of longer than two months due to stable, non-progressive disease, even with minimal exudation. Despite the presence of active choroidal neovascularization lesions, VA showed significant improvement for up to three years postoperatively. Moreover, the postoperative recurrence rate of exudation decreased. Previous studies have either reported no change [13,19] or marginal reduction [15] in the number of injections administered before and after surgery, with follow-up periods of several months to one year.

We have complemented some previously reported predictive factors of visual outcomes [17,18] by following up eyes under active anti-VEGF treatment for nAMD for over three years. It may be difficult to obtain sufficient visual outcomes after cataract surgery if the duration of AMD spans several years, with extremely poor preoperative vision. The exudation-free period and postoperative presence of exudation had no discernible effects on the visual outcome. More frequent postoperative visits are recommended to evaluate exudation recurrence in patients with prolonged AMD and a short exudation-free period. Unexpectedly, the frequency of preoperative injections and the combination of injections with cataract surgery did not affect AMD progression postoperatively.

This study had some limitations. First, the study design was retrospective. Second, we included eyes with heterogenous AMD subtypes, treated with a variety of anti-VEGF agents and treatment regimens. However, this diversity could also be viewed as a strength of the generalizability of our results. The absence of a matched control group that received anti-VEGF therapy and no cataract surgery is also a limitation.

The present study also has several strengths. First, a detailed analysis of pertinent data was performed, including VA and OCT results, before (32.7 months) and after cataract surgery (up to 49.6 months). Second, we identified the long-term conditions of nAMD and visual improvements after cataract surgery in eyes with exudative nAMD undergoing active treatment. Third, the preoperative VA, nAMD duration, and exudation-free period were identified as predictive factors under these clinical conditions.

## 5. Conclusions

In conclusion, we suggest that cataract surgery could be performed safely in eyes with exudative nAMD receiving active anti-VEGF treatment. The different exudation-free periods and nAMD durations may provide clinicians with useful information for determining the optimal timing of cataract surgery. Preoperative VA is the most effective predictive factor for determining the long-term postoperative visual outcome after cataract surgery. Long-term clinical trials conducted under controlled conditions are needed to validate the conclusions of this study.

## Figures and Tables

**Table 1:** Characteristics of patients who underwent cataract surgery during active treatment for nAMD.

	Total	*p*-Value
**Basic characteristics (patients: *n* = 61)**		
Age, years	74.0 ± 7.6	
Male/Female, *n*	44/17	
Co-morbidities, *n* (%)		
Hypertension	17 (27.9)	
Diabetes	13 (21.3)	
**Before cataract surgery (eyes: *n* = 65)**		
AMD duration, months	32.7 ± 32.9	
AMD subtype, *n* (%)		
MNV Type I	37 (57.0)	
PCV	17 (26.2)	
MNV Type II	8 (12.3)	
MNV Type III	3 (4.6)	
Regimen of anti-VEGF injections		
Pro re nata	43 (66.2)	
Treat-and-extend	22 (33.8)	
Total number of anti-VEGF injections	14.2 ± 13.6	
Interval from the last injection to surgery, days ^a^	70.8 ± 55.8	
Exudation-free period, days	23.0 ± 47.1	
Anti-VEGF injection interval, days/injection ^b^	66.5 ± 38.9	
**Cataract operation (eyes: *n* = 65)**		
Presence of exudation, *n* (%)	46 (70.8)	
Phacoemulsification with IOL implantation, *n* (%)	32 (49.2)	
Phacoemulsification with IOL implantation plus anti-VEGF, *n* (%)	33 (50.8)	
**After cataract surgery (eyes: *n* = 65)**		
Follow-up period, months	49.6 ± 37.8	
Total number of anti-VEGF injection	12.4 ± 12.9	
Interval to the subsequent injection, days ^a^	88.1 ± 41.9	0.21
Anti-VEGF Injection interval, days/injection ^b^	228.0 ± 39.2	0.001

Values are expressed as mean ± standard deviation (SD), or number of subjects and percentage, as appropriate. ^a,b^ Used for paired *t*-test comparing intervals before and after surgery. nAMD: neovascular age-related macular degeneration; VEGF: vascular endothelial growth factor; BCVA: best-corrected visual acuity; logMAR: logarithm of the minimal angle of resolution; PCV: polypoidal choroidal vasculopathy; MNV: macular neovascularization; IOL: intraocular lens.

**Table 2:** Analysis of prognostic factors associated with postoperative BCVA (logMAR) at one year.

Factors	Simple		Multiple	
	Coefficient (SE)	*p*-Value	Coefficient (SE)	*p*-Value
Age	0.001 (0.004)	0.76		
Preoperative BCVA ^a^	0.68 (0.093)	<0.001	0.50 (0.10)	<0.001
AMD duration ^a^	0.006 (0.002)	0.001	0.004 (0.001)	0.003
AMD subtype	0.07 (0.098)	0.48		
Exudation-free period	−0.003 (0.001)	0.61		
Presence of exudation at surgery	0.15 (0.12)	0.22		
Injection interval, preoperative	0.001 (0.001)	0.39		
Combined anti-VEGF during cataract surgery	−0.08 (0.048)	0.11		
Interval to subsequent recurrence	0.001 (0.001)	0.22		

^a^ Factors with the lowest *p*-values in a simple linear regression analysis (i.e., *p* < 0.10) were included in a multiple regression analysis. SE: standard error; BCVA: best-corrected visual acuity; logMAR: logarithm of the minimal angle of resolution; AMD: age-related macular degeneration; VEGF: vascular endothelial growth factor.

**Table 3:** Analysis of prognostic factors associated with the interval of postoperative anti-VEGF injections.

Factors	Simple		Multiple	
	Coefficient (SE)	*p*-Value	Coefficient (SE)	*p*-Value
Age	−1.51 (6.25)	0.49		
Diabetes	−14.5 (94.7)	0.74		
AMD duration ^a^	−3.50 (1.60)	0.034	−3.38 (1.50)	0.028
AMD subtype	0.20 (1.50)	0.14		
Exudation-free period ^a^	1.91 (1.02)	0.010	2.77 (0.88)	0.003
Presence of exudation at surgery	−118.7 (88.3)	0.18		
Injection frequency, preoperative	1.68 (1.20)	0.17		
Combined anti-VEGF during cataract surgery	27.1 (90.3)	0.67		
Interval to subsequent recurrence	0.05 (0.8)	0.71		

^a^ Factors with the lowest *p*-values in a simple linear regression analysis (i.e., *p* < 0.10) were included in a multiple regression analysis. SE: standard error; AMD: age-related macular degeneration; VEGF: vascular endothelial growth factor.

## Data Availability

The datasets used and analyzed in the current study are available from the corresponding author on reasonable request.
